# Random number generation and the ability of mentally reconstructing context in patients with organic amnesia

**DOI:** 10.1590/1980-5764-DN-2021-0021

**Published:** 2022

**Authors:** Nariana Mattos Figueiredo Sousa, Ivanda de Souza Silva Tudesco, Silvia Adriana Prado Bolognani, Silmara Batistela, Orlando Francisco Amodeo Bueno

**Affiliations:** 1 Universidade Federal de São Paulo, Departamento de Psicobiologia, São Paulo SP, Brazil.; 2 Faculdade Censupeg, Programa de Pós-Graduação em Neuropsicologia, Joinville SC, Brazil.; 3 Centro Paulista de Neuropsicologia, São Paulo SP, Brazil.; 4 Centro Paulista de Neuropsicologia, São Paulo SP, Brazil.

**Keywords:** Amnesia, Memory, Short-Term, Memory, Episodic, Executive Function, Amnésia, Memória de Curto Prazo, Memória Episódica, Função Executiva

## Abstract

Studies investigating amnesic patients have shown the involvement of the medial temporal lobe during working memory (WM) tasks, especially when multiple items or features have to be associated. However, so far, no study has examined the relationship between episodic memory and WM components in patients with amnesia for comprehensive neuropsychological evaluation.

**Objective:**

The objective of this study was to investigate whether the null retention relates to deficits in the episodic buffer (EB) or the central executive (CE) components of WM.

**Methods:**

This study included 15 amnesic patients with mixed etiologies and 13 matched healthy controls. These 15 amnesic patients with mixed etiologies were divided into two subgroups: NUL subgroup (n=7) patients whose raw score was 0 (zero) on the Logical Memory delayed recall test and MOR subgroup (n=8) patients who recalled at least 1 item. The EB was assessed by complex span tasks, and the CE was assessed by random number generation (RNG) test.

**Results:**

EB tasks were impaired in both subgroups compared with controls. RNG was impaired in NUL (p=0.03), but not in MOR (p=0.99), subgroup.

**Conclusions:**

CE impairment hampers the retrieval mode action, preventing it from initiating the mental reconstruction of the context in which the to-be-remembered information was presented minutes ago.

## RESUMO.

Estudos que investigaram pacientes amnésicos demonstraram envolvimento do lobo temporal medial durante tarefas de memória de trabalho, especialmente quando vários itens ou características devem ser associados. No entanto, até o momento, não há estudos que tenham examinado a relação entre memória episódica e os subcomponentes da memória de trabalho em pacientes com amnésia por meio de avaliação neuropsicológica ampla.

**Objetivo:**

Investigar se a retenção nula está relacionada a déficits no *buffer* episódico ou nos componentes do executivo central da memória operacional.

**Métodos:**

Quinze pacientes amnésicos com etiologias mistas foram divididos em dois subgrupos: subgrupo NUL (n=7), de pacientes cuja pontuação bruta foi 0 (zero) na memória lógica tardia, e subgrupo MOR (n=8), de pacientes que recordaram pelo menos um item; além de 13 controles saudáveis pareados. O retentor episódico foi avaliado por tarefas de *spam* complexo e o executivo central com geração aleatória de números.

**Resultados:**

As tarefas do retentor episódico estavam prejudicadas em ambos os subgrupos em comparação com os controles. O teste de geração aleatória de números foi prejudicado em NUL (p=0,03), mas não no subgrupo MOR (p=0,99).

**Conclusões:**

O comprometimento do executivo central dificulta a ação do modo de recuperação, impedindo-o de iniciar a reconstrução mental do contexto em que a informação a ser lembrada foi apresentada, minutos antes.

## INTRODUCTION

The term amnesia refers to a pathological mental state in which memory and learning are affected, in greater proportion than other cognitive functions, in a patient without altered level of consciousness^1^. In some amnesic patients, memory seems to vanish as soon as the focus of attention diverts from the just experienced episode ^2,3^.In others, however, the recently acquired memory seems to resist some time further, allowing them to manipulate the new information even under conditions of external distraction, as in a report of patients with global amnesia who were able to remember short stories immediately after their presentation ^4^. Baddeley and Wilson^4^ interpreted the ability of amnesic patients to immediately remember short stories as evidence that the immediate recall of a text demands efficiency of the central executive (CE) and also the involvement of the episodic buffer (EB). However, Gooding et al.^5^ did not obtain a positive correlation between the scores of immediate recollections of stories and measures of executive functioning. Complex span tasks require the simultaneous storage and manipulation of information that far reaches the range of short-term memories usually assessed by means of simple span tasks.

Some studies show that executive aspects, working memory (WM) components, may be associated with the formation of episodic memories, besides participating in retrieval and encoding stages, as well as medial temporal lobe (MTL) is critical for supporting WM even for a single item and that it contributes selectively to sensing-based discriminations^6,7^. Functions related to CE, such as organization, attention, and mental manipulation of information, are important for some mnemonic stages, such as encoding and decoding; however, studies investigating the relation between WM components, assessed through sensitive tests for EB and CE, and episodic memory measures are necessary for elucidating this possible interaction.

Olson et al.^8^ noted that, until quite recently, episodic memory and WM were considered independent from each other, the former supported by MTL and related structures, and WM relied on prefrontal cortex (PFC) and regions of the parietal lobe. If this were the case, WM would be irrelevant for episodic memory performance in amnesic patients. Indeed, the EB was suggested to serve as an interface between WM and episodic memory^9,10^.

Notwithstanding the experimental evidence pointing to the relations between PFC and MTL, the relevance of different WM components for episodic memory formation in patients with organic amnesia is quite understudied.

The aim of this study was to investigate whether the null retention, assessed through an episodic memory test, would be related to deficits in the EB or the CE components of WM.

## METHODS

### Participants and recruitment

This study included 15 organic amnesic patients with varied etiologies and 13 healthy controls. All patients presented a profound anterograde amnesia attested by clinical diagnosis and neuropsychological testing. The exclusion criteria were diagnosis of dementia, other neurological diseases, mood or anxiety disorders, history of alcohol/drug abuse, and uncorrected sensory deficits (which were investigated by clinical interview and specific scales for anxiety and depression). For this, scales to assess mood, anxiety, and family interview were used. As a criterion for being included in the whole amnesic sample, each patient had to have a raw score of at least 2 standard deviations (SDs) below the control non-amnesic group in the delayed Logical Memory (LM) test. Patients must also present preserved performance on implicit memory tests.

The clinical sample was stratified into two subgroups: (1) amnesic patients who scored zero in the delayed prose recall task (NUL subgroup; n=7) and (2) amnesic patients who presented a raw score more than zero (MOR subgroup; n=8). The patients were referred by the Centro Paulista de Neuropsicologia (CPN-Reab) e Hospital São Paulo and Universidade Federal de São Paulo (UNIFESP). The control group participants were recruited through the community and matched for sex, age, and education for which the same exclusion criteria were applied. The study protocol was approved by the research ethics committee of the Universidade Federal de Sao Paulo.

### Neuropsychological assessment

#### Episodic memory tests

*Story recall:* The LM test ^11^ was used for episodic memory evaluation with two narratives (A and B) of 25 items each; the evaluator reads them one at a time, with the subject having to recall immediately after the presentation and after 30 min (late recall). The score is the sum of the number of items evoked, immediately and later.

*Visual reproduction* ^11^: This aims to assess immediate and delayed episodic memory for visual content. The material consists of four stimulus cards, with different geometric shapes on them. Each card is presented for 10 s and, during this time, the subjects will limit themselves to observe the figure. Immediately after exposure, the subject shall reproduce the stimulus observed. Later recall of figures is performed for 30 min after exposure. The score is the total of figure characteristics recalled, immediately and later.

*Rey–Osterrieth Complex Figure – copy and memory* ^12^: It consists of a complex geometrical figure that comprises a large rectangle, the horizontal and vertical bisectors, two diagonals, and additional geometric details inside and outside the large rectangle. After copying, the subjects are asked to draw the figure based on their immediate recall. After 30 min, again, the drawing is recalled. Correction is done based on tracing precision and adequate location, immediately and later.

#### Working memory tests

*Random number generation (RNG)* ^13^:It is used as a measure of the WM CE^14–16^. RNG is a specific form of the task, in which the subject generates a random sequence of numbers from 1 to 9 paced by a tone. The random requirement compels attentive control in order to perform a series of executive function operations, such as inhibiting habitual sequences, using short-term memory to remember the last items, updating the number sequence, set shifting, and monitoring the responses. PFC areas and parietal regions underline these functions^17,18^. More specifically^16^, it demonstrated that PFC is engaged during the performance of RNG, thus being an instrument of brief and effective application to clinically assess changes in the frontal lobe, as well as a valuable instrument to evaluate executive processes after brain injury.

*Operation span (OSPAN)* ^19,20^: It evaluates the capacity of the WM EB^9^. It is commonly used to assess individual differences of WM capacity^21^, trying to remember words while solving mathematical operations^22^.The score obtained in this test reflects the storage capacity of the EB^15,23,24^. In contrast, random generation tasks are suitable instruments to assess the CE because they implicate the majority of functions ascribed to this WM component.

*Counting span (OSCAN)* ^22^: It evaluates the functioning of the EB and consists of counting items instead of words. It consists of sequences of screens in which blue circles, white circles, and white squares appeared distributed on a black background. White circles were used as counting targets and ranged in numbers from 3 to 9; the other figures were used as distracting stimuli. The participant had to count the number of white circles, without pointing and checking the total in loud voice. At the end of each series, a screen with a question mark appeared, and the subject had to say the number of white circles counted in each of the previous screens, without necessarily being in the order in which they appeared. This test had sequences varying between two screens to count (span 2) up to six screens (span 6). The number of stimuli to be counted on each screen varied randomly. The storage component consisted of the result of each count in the processing component. A span 3 training was performed.

*Digit span* ^25^: This task is subdivided into two parts: subjects are asked to repeat in the same order (forward) and another in reverse order (backward) a series of digits recited orally by the examiner. This task is subdivided into two parts, such as original order and reverse order. The score is the number of digits in the maximum sequence repeated correctly. In reverse order, the test format is the same, but the subject shall remember the numbers in reverse, after two examples with two and three numbers. The test objectives in original and reverse order are to assess the storage and reverberation capacity in verbal immediate memory (phonological loop) and the capacity to maintain and manipulate information (CE).

#### Mood and anxiety scales

*STAI-Trait and State* ^26^: It is a self-report scale that measures two elements of anxiety. According to this inventory, the state scale requires the participant to describe how they feel “now, in this moment” in relation to 20 items presented on a four-point Likert-type scale, ranging from 1=absolutely not, 2=a little, 3=a lot, and 4=very much. Similarly, the trait scale also consists of 20 items, but the participant is instructed to respond as “they usually feel,” according to a new four-point Likert-type scale, ranging from 1=almost never, 2=sometimes, 3=often, and 4=almost always. The total score varies between 20 and 80 points, with higher values indicating higher levels of anxiety.

*Beck Depression Inventory (BDI)* ^27^: It is a self-report questionnaire, consisting of 21 multiple-choice items measuring the intensity of depressive symptoms. It consists of several items related to depressive symptoms, such as hopelessness, irritability, and cognitions, such as guilt or feelings of being punished, as well as physical symptoms such as fatigue, weight loss, and decreased libido. Depression severity is measured through scores: mild (12–19), moderate (20–35), and severe (36–63).

#### General level of intelligence

*Raven’s Progressive Matrices – General Scale* ^28^: It assesses general intelligence, more specifically, the subject’s ability to deduce relations. It includes 60 items divided into five sets of 12 items ordered by the level of difficulty. Various items consisted of nonverbal material. This scale has an application notebook where five subsets of items (i.e., A, B, C, D, and E) were represented. A raw score was used.

### Procedure

Participants were assessed individually in 4–7 sessions for one and a half hours. The test sequence was randomized, and fatigue was accounted for. Mood was assessed using the BDI and the Trait Anxiety Inventory (TAI), respectively, for subjective report of depression and anxiety symptoms. Nonverbal intelligence was evaluated using Raven’s Progressive Matrices.

### Statistical analyses

Descriptive analyses included the presentation of means and SD in tables. For inferential analyses, demographic data and cognitive tests’ scores were the variables treated (analysis of variance or ANOVA), and the Amnesic and Control groups and MOR and NUL subgroups were considered as factors. Post hoc Tukey Honest Significant tests were used when pertinent. Due to small sample size, Bayes factor analysis was included to verify the confidence of these results.

The level of significance was 5%. All analyses were performed using *Statistical Package for the Social Sciences* (SPSS) software, version 22.0.

## RESULTS

### Demographic characteristics and clinical data

Amnesia’s etiology, age, and schooling of patients are shown in Table 1. The age ranged from 22 to 67 years and schooling varied from 9 to 18 years. Etiology was also varied within the sample, with the majority from traumatic brain injury (TBI).

**Table 1:** Etiology of amnesia, age, and schooling of patients.

Amnesic patients	Age	Years of study	Etiology
ST	49	16	Stroke
SC	32	12	Stroke
AF	45	11	TBI
MB	43	15	Encephalitis
RT	44	18	Anoxia
AC	36	9	Tumor^a^
JP	67	16	Anoxia
MQ	60	18	Stroke
ED	22	9	TBI
GB	52	12	TBI
EM	24	11	Encephalitis
EC	35	12	TBI
HP	59	16	TBI
LM	29	13	TBI
JR	62	16	Hydrocephalus

In the left column are shown the initial letter of patient’s names. TBI: traumatic brain injury

a brain tumor.

Table 2 shows the demographic characteristics (i.e., age, schooling, and gender) and clinical data (i.e., subjective symptoms of depression and anxiety) between amnesic patients and control group. There is no difference between age (p=0.62) and years of schooling (p=0.53), as the sample was paired for such characteristics. The measures of depression (p=0.5) and anxiety (p=0.79 for anxiety state and p=0.98 for anxiety trait) did not differ between groups. Nonverbal intelligence measures did not differ between groups (p=0.22) (Table 2).

**Table 2:** Demographic and clinical characteristics and subjective symptoms of depression and anxiety of the whole sample.

		Amnesic patient		Control group		
		n		n		
Gender						
	Female	4		5		
	Male	11		8		
		M	SD	M	SD	p-value
Age		43.93	14.20	40.85	12.88	0.62
Schooling		13.60	3.02	12.77	2.49	0.53
Raven		38.87	10.31	43.77	7.34	0.22
BDI		5.73	2.87	4.69	3.35	0.5
STAI-S		36.87	5.48	36.46	4.84	0.79
STAI-T		39.60	5.87	39.31	6.59	0.98

Schooling and years of injury are shown in years. Raven: Raven’s Progressive Matrices – Nonverbal Intelligence Measure; BDI: Beck Depression Inventory; STAI-S: Trait Anxiety Inventory – State; STAI-T: Trait Anxiety Inventory – Trait.

The patients’ sample was divided into two subgroups, one composed of amnesic patients who had zero as raw score in the delayed LM task (NUL subgroup; n=7) and the other comprised of patients with more than zero raw score (MOR subgroup; n=8). Thus, the results of each amnesic subgroup are presented, compared with the control group.

ANOVAs including the control group (see Table 3) revealed differences among groups. The control group performed better than both amnesic subgroups in immediate (F_2,57_=25.53; p<0.001) and delayed recall (F_2,57_=91.46; p<0.001) of LM. In Rey–Osterrieth Complex Figure immediate recall (F_2,57_=23.20; p<0.001) and delayed recall (F_2,57_=19.89; p<0.001) and Visual Reproduction in immediate recall (F_2,57_=25.35; p<0.001) and delayed recall (F_2,57_=67.02; p<0.001).

**Table 3:** Performance in episodic memory task of amnesic subgroup (MOR and NUL) and control group.

		MOR (n=8)		NUL (n=7)		Control group (n=13)		F_2,25_	MOR ×NUL	Control×MOR	Control×NUL	MOR× NUL
Episodic memory tests		M	SD	M	SD	M	SD		p-value	Tukey test	Tukey test	Tukey test
Verbal memory												
	LM immediate	13.75	6.32	9.29	7.52	29.08	6.13	25.53	<0.001	0.001	0.003	0.42
	LM delayed	4.13	2.64	0.00	0.00	26.77	6.67	91.46	<0.001	0.001	0.001	0.26
Visual memory												
	ROCF immediate	7.68	5.46	4.71	2.95	18.96	5.45	23.20	<0.001	0.004	0.001	0.51
	ROCF delayed	7.75	5.58	3.85	3.13	18.19	5.88	19.89	<0.001	0.001	0.002	0.36
	VR immediate	26.25	4.89	25.71	3.72	35.61	2.36	25.35	<0.001	0.001	0.001	0.95
	VR delayed	10.75	5.47	5.57	3.69	30.84	5.66	67.02	<0.001	0.001	0.001	0.17

This table shows the performances (mean and SD) of the sample (MOR, NUL, and control group) in episodic memory test. Also, it shows the comparison between amnesic subgroups, MOR and NUL, MOR subgroup and control group, and NUL subgroup and control group. LM: Logical Memory; ROCF: Rey-Osterrieth Complex Figure; VR: visual reproduction.

Regarding WM tasks (Table 4), the control group performed better than MOR subgroup in forward (which measures phonological loop) and backward digit span task [(F_2,57_=6.95; p<0.001) and (F_2,57_=5.73; p<0.001), respectively], but there were no differences with NUL subgroup.

**Table 4:** Performance in working memory task of amnesic subgroups (MOR and NUL) and control group.

Working memory test	MO R (n=8)		NUL (n=7)		Contro l group (n=13)		F_2,25_	MOR× NUL	Contr ol×MOR	Contr ol×NUL	MOR× NUL
Digit span	M	SD	M	SD	M	SD		p-value	Tukey test	Tukey test	Tukey test
Forward	4.50	1.3 0	4.85	0.6 9	6.15	1.0 6	6.95	0.003	0.01	0.07	0.80
Backward	3.12	1.3 5	4.0	1.5 2	5.0	1.0	5.73	0.008	0.01	0.30	0.40
OSPAN	18.1 2	4.3 2	16.5 2	4.3 9	29.2	4.8 2	23.1 6	<0.000 1	0.002	0.002	0.80
CSPAN	41.0	7.2 8	40.0	6.0 6	52.30	3.6 8	16.0 0	<0.000 1	0.001	0.009	0.93
RNG	0.36 4	0.0 5	0.51 4	0.1 7	0.365	0.0 7	5.46	0.01	0.99	0.03	0.03

This table shows the performances (mean and SD) of the sample (MOR, NUL, and control group) in working memory test. Also it shows the comparison between amnesic subgroups, MOR and NUL, MOR subgroup and control group, and NUL subgroup and control group. OSPAN: Operation Span: Operation Span Task; CSPAN: Counting Span: Counting Span Task; RNG: Random Number Generation Task.

In OSPAN (WM capacity), the control group performed better than both amnesic subgroups — MOR (F_2,57_=23.26; p=0.002) and NUL (F_2,57_=23.26; p=0.002). The same result was observed in CSPAN (which also measures WM capacity), with the control group performing better than MOR (F_2,57_=16.00; p=0.001) and NUL (F_2,57_=16.00; p=0.009). No differences were observed between MOR and NUL in both tasks.

The control group performed better than NUL in RNG task, which is a CE measure (F_2,57_=5.46; p=0.03). In this same task, MOR performed better than NUL (F_2,57_=5.46; p=0.03), but there were no differences between control group and MOR.

The Bayes factor analysis showed the same pattern of result, as can be seen in Table 5.

**Table 5:** Bayesian analysis of variance.

		All		Cont vs MOR		Cont vs NUL		MOR vs NUL	
		p-value	BF_10_ (Bayes Factor)	p-value	BF_10_ (Bayes Factor)	p-value	BF_10_ (Bayes Factor)	p-value	BF_10_ (Bayes Factor)
LM Immediate		<0.001	5.798e-5	<0.001	606	<0.001	2361	0.42	0.724
LM Delayed		<0.001	4.891e-10	<0.001	312245	<0.001	1.432e+6	0.26	24.8
ROCF Immediate		<0.001	1.172e-4	<0.001	110	<0.001	2343	0.51	0.742
ROCF Delayed		<0.001	3.529e-4	0.001	38	<0.001	1133	0.36	1.006
VR Immediate		<0.001	5.916e-5	<0.001	1353	<0.001	11642	0.95	0.445
VR Delayed		<0.001	1.157e-8	<0.001	51968	<0.001	1.640e+6	0.17	1.677
Digit Span									
	Forward	0.003	0.076	0.01	8.5	0.07	5.2	0.80	0.5
	Backward	0.008	0.147	0.01	19.4	0.30	1.2	0.40	0.7
	OSPAN	<0.001	1.168e-4	<0.001	406	<0.001	756	0.80	0.5
	CSPAN	<0.001	0.001	0.001	146	<0.001	691	0.93	0.45
	RNG	0.01	0.2	0.99	0.4	0.03	3.8	0.03	2.2

Bayer factor: Evidence category>100=Extreme evidence for H1; 30-100=Very strong evidence for H1; 10-30=Strong evidence for H1; 3-10=Moderate evidence for H1; 1-3=Anecdotal evidence for H1; 1=No evidence; 0.33-1=Anecdotal evidence for H0; 0.10-0.33=Moderate evidence for H0; 1=No evidence; 0.33-1=Anecdotal evidence for H0; 0.10-0.33=Moderate evidence for H0.

## DISCUSSION

The aim of this study was to investigate whether deficits in long-term retention in amnesic patients, assessed through an episodic memory test, would be related to WM subcomponents. As mentioned in the literature, this population shows impairment in the storage of information following distracting activities (delayed recall), while measurements of short-term, implicit, and intellectual memory are preserved. Moreover, there is a selective mnemonic difficulty, as they are able to show better performance after repetitive activities, which follow constant rules, as it was observed in the implicit memory tests (procedure and pre-activation memory).

The main finding was that clinical subgroups differed in the RNG task, with the MOR patients showing a better performance than NUL patients, and similar to the control group, suggesting that their executive capacities, such as inhibitory control, concentration, and updating of information, are not similar in the different clinical groups. As both subgroups achieved a lower performance in the immediate-phase recall, it seems unlikely that immediate recall is affected by the CE component of WM.

In both amnesic subgroups, EB functioning and immediate LM were diminished. Deficits in immediate episodic memory recall may be due, at least in part, to a dysfunction of the EB. Previous authors^10,29,30^ have suggested that EB would fill the gap between stimuli input for temporary, but sufficient time, to consolidating take place in the MTL and contribute to the formation of episodic memory.

We propose, instead, that immediate recall depends on the capacity to maintain it for a brief period (longer than the capacity of STM) with the help of the EB that, in turn, depends partially on the proper functioning of the memory circuit.

Preserved CE functions, in turn, would permit a sufficient level of attention control of the EB to maintain its capacity for immediate episodic recall, but not at a sufficient level to allow consolidation in long-term memory.

Therefore, recollection of episodic memory involves the reconstruction of lived experiences. In immediate recall of episodic memory tests, the physical context (the room, the furniture, the presence of the experimenter, and so on) varied very little from the acquisition moment to the retrieval moment, that is, the mental state remains still close and similar to that of acquisition. But the internal subjective context moves on to a further moment, more distinct from the former, because the temporal lag is filled up by some conversation, other instructions, tasks, feelings, thoughts, postures, and so on. In cued recall, external cues guide subject’s mind to reconstruct the temporal and spatial context in which the episode occurred. However, when no appropriate external cues are available, the subject must internally generate appropriate retrieval cues^31^. To do this, the subject must bring back a mental state that lies on the background of conscious attentional focus, which holds a fragment of one’s personal past, the so-called retrieval mode, as Tulving denominated it^32^.

Retrieval mode guides subsequent events to serve as plausible internal and external retrieval cues for consciously remembering a particular past event^33^.

Engagement of PFC and connected networks is important for entering into retrieval mode and reinstalling an updated context by the cues available at the present moment^33–38^, a process probably precluded in the NUL patients. We interpret the complete failure in scoring in the delayed recall to a putative prefrontal–parietal network damage, since the RNG task critically involves these cerebral areas and it was impaired in them, RNG critically depends on PFC to accomplish the task.

The scores of immediate recalls of the two amnesic subgroups did not differ between them but were equally worse than that of controls. The same pattern was seen regarding complex span tasks (i.e., OSPAN and OSCAN). Assuming that the subjective context is not likely to change very much between acquisition and testing, retrieval mode is not critically needed in immediate recall, with EB capacity provisionally maintaining the recently acquired information to be manipulated. Studies have identified the MTL as a possible candidate to be the neural basis of EB^39^. Therefore, the impaired EB capacity in amnesic patients can be attributed to MTL damage. If this line of reasoning is correct, one may consider the EB as an intermediate memory or interface between WM and long-term memory (LTM), as suggested by Quinette et al. and Greenberg et al^10,40^.

We propose to interpret this situation as not a simple case of degree of memory loss amnesic individuals present, attributed to MTL lesions extension, but rather to a retrieval mode failure that prevents reinstatement of an updated context by cues supplied at the moment of recollection.

Our results suggest that impairment of CE in deep amnesic patients is related to an extratemporal lobe damage, constituting a special subpopulation bringing a more profound and complex memory impairment. An extrahippocampal origin of the RNG impairment in NUL patients is strongly supported by recent findings^24^ in which neither left nor right unilateral hippocampal resection in MTL epileptic patients had an effect on RNG, although left resection impaired OSPAN task performance. Knowing in advance, by using a quick and easy-to-apply task (as is the case of RNG test), if the CE is or not impaired would contribute to a more comprehensive assessment of the patient’s pathological condition.

The present finding is relevant as it permits to identify in advance the amnesic patients who can focus and maintain enough attention. This distinction of groups might help in the planning of rehabilitation strategies, with specific techniques for those patients with the additional focus/attention deficits.

As immediate LM test was also impaired, Baddeley and Wilson’s^4^ suggestion that immediate prose recall depends on the capacity of the EB plus some level of CE functioning cannot be ruled out. However, it should be noted that a gradual dependency of EB on CE was not detected.

There are no studies in the literature showing the relationship between these WM components and information retention based on episodic memory tests. Thus, we cannot compare this study with previous ones, which increases the importance of the data obtained, despite the small sample size.

Therefore, we suggest that CE abilities are critical to assure some level of delayed recall, although a low one compared with non-amnesic subjects. One possibility is that preserved executive control of the MOR patients directs enough attentional resources to permit some level of consolidation.

Despite a profound amnesia, some patients are able to achieve long-term retention performance (albeit in a much lower level than non-amnesic ones). As lesion to PFC impairs RNG performance and this region is often injured in amnesic patients, it is likely that the NUL subgroup had PFC damage in addition to MTL areas. PFC is known to cause executive function deficits, which lead to difficulties in initiation and information generation, planning, and organization^40^. A cognitive model created by Tulving is proposed to account for the present findings^32^. Retrieval mode is necessary to initiate mental reconstruction of the context in which the to-be-remembered information is presented.

This study has some limitations, such as the absence of imaging exams, which would help to understand the relations with topographic mechanisms of the neurological lesions; in addition, the patients had varied neurological lesions, thus not being a homogenous sample; and the sample was small. However, studies with amnesic patients generally had a small number of patients (n<30). Still, the findings support important theoretical considerations, and also possibly clinical implication, as there are no similar studies in the literature.

These data suggest that the reduced WM capacity compromises the formation of episodic memory in amnesic patients. Alternatively, the reduction and impairment of episodic memory may suggest less ability to establish relationships between unrelated items and between these and the context.

This result makes it possible to think about rehabilitation strategies, since amnesic patients are able to focus and sustain attention, however, when the material does not involve speed of mental processing and more complex materials, that is, with a greater amount of information.

Subsequent studies, with larger samples, using brain images and more restricted hippocampal lesions are important for a better understanding of the relationships between the episodic and components of WM systems.
