# Erratum

**DOI:** 10.1590/1980-5764-DN-2021-0021erratum

**Published:** 2022-08-05

In the article “Random number generation and the ability of mentally reconstructing context in patients with organic amnesia”, with DOI code number 10.1590/1980-5764-DN-2021-0021, published in the Dement Neuropsychol 2022 March;16(1):19-27, on page 19:

**Where it was written:**

^4^Centro Paulista de Neuropsicologia, São Paulo SP, Brazil.

**Should read:**

^4^Centro Universitário São Camilo, São Paulo SP, Brazil.

